# Exploring Type IIIb Endoleaks: A Literature Review to Identify Possible Physical Mechanisms and Implications

**DOI:** 10.3390/jcm13154293

**Published:** 2024-07-23

**Authors:** Tuna Aras, Mahmoud Tayeh, Adel Aswad, Mohamed Sharkawy, Payman Majd

**Affiliations:** 1 Vascular Surgery Department, EVK Bergish Gladbach Ferrenbergstraße, 51465 Bergisch Gladbach, Germany; t.mahmoud@evk.de (M.T.); p.majd@evk.de (P.M.); 2 Al-Qassimi Teaching Hospital and Cardiac Centre, University of Sharjah, Sharjah P.O. Box 3500, United Arab Emirates; vascular1984@gmail.com; 3 Cairo University Hospitals, Cairo Governorate 4240310, Egypt; sharkawy54@me.com

**Keywords:** endoleak type IIIB, EVAR, abdominal aortic aneurysm

## Abstract

Endoleaks are common complications following endovascular aneurysm repair (EVAR). They can be classified into low-pressure and high-pressure endoleaks. High-pressure endoleaks, which include Type I and Type III endoleaks, pose a significant risk of rupture and require urgent treatment. The aim of our study is to review published case reports and case series to assess the impact of Type IIIb endoleaks in EVAR and to identify possible mechanisms contributing to these endoleaks. This review targeted case reports and case series published between January 1998 and December 2022. A total of 62 case reports and case series were identified, encompassing 156 patients with Type IIIb endoleaks. Data collection was performed by three consultants who thoroughly discussed each report before registering it into an analyzable data set. Our analysis revealed that, beyond material imperfections, certain endograft configurations or conformations, endograft redundancy, and the physical forces acting on the grafts may lead to increased stress on specific parts of the endografts, potentially exceeding their design limits. Factors contributing to redundancy and unfavorable conformation of the endograft include secondary interventions for any cause (such as other types of endoleaks), EVAR performed outside the instructions for use (IFUs), endograft migrations, or larger initial aneurysm diameter.

## 1. Introduction

An endoleak can be defined as the continuous perfusion of the aneurysm sac following aortic endografting. Despite significant advancements in imaging and post-processing technologies, which have facilitated improved sizing and planning, and advancements in material technology that have led to the development of more durable endografts, endoleaks continue to negatively impact treatment outcomes. Approximately 30% of patients develop an endoleak endovascular aneurysm repair (EVAR) [1]. Among cases with endoleaks, 12–20% require reintervention [2,3]. Additionally, ruptures occur in 1–2% of cases within five years following aortic endografting [4,5].

Endoleaks are generally classified into five categories. Type I endoleaks occur due to an inadequate seal between the aorta and the endograft at either the proximal or distal attachment site. Type II endoleaks involve retrograde filling of the aneurysm sac and can arise from single or multiple vessels. Type III endoleaks result from component separation or a failure in the endograft’s integrity, such as tears. Type IV endoleaks are attributed to the porosity of the grafts, a condition now extremely rare thanks to advancements in the latest generation of grafts. Type V endoleaks, also known as endotension, are characterized by aneurysm sac expansion post-endografting, occurring without any detectable leakage [6,7].

Type I and Type III endoleaks can be categorized as high-pressure endoleaks, which may occur during graft deployment or during follow-up. Immediate treatment is imperative upon detection due to the exposure of the aneurysm sac to systemic pressure. Furthermore, a study indicates that only 20% of Type IIIb endoleaks are detectable with computed tomography angiography (CTA) [8], suggesting that the reported incidence of 2–4% may be underestimated [9,10,11]. Moreover, there are case reports indicating the detection of fabric tears post open surgical conversion in patients exhibiting sac expansion without detectable leakage on CT angiograms. This observation raises concerns that a certain proportion of Type V ‘Endotension’ cases may be attributable to microfabric tears. This scenario presents serious concerns, particularly when patients do not exhibit aneurysm sac regression.

In our review, we sought to identify case series and reports that elucidate the circumstances leading to Type IIIb endoleaks, their possible mechanical and physical causes, and, in the last part, postulate potential predictive factors.

## 2. Materials and Methods

An electronic search of the PubMed, Medline, and Cochrane databases was conducted to obtain reports and series exclusively concerning Type IIIb endoleaks. This search targeted case reports and case series of patients treated between January 1998 and December 2022. Data collection was performed by three consultants who thoroughly discussed each report before registering it into an analyzable data set. A standardized form was employed for data collection. The extracted data included study characteristics (year of publication, number of cases), endograft characteristics (brand and model), detection time of endoleak post initial implantation, presence of rupture at presentation, presence or absence of diameter increase at presentation, location of the leak, treatment strategies and modalities, and mortality rates. For further analysis, to determine associations and precision, the Chi-Square Test was used to assess the overall association between categorical variables, such as diagnostic methods and locations of tears. Fisher’s Exact Test was applied for 2x2 contingency tables where expected cell counts were less than 5. To accommodate the uneven distribution of data, the Kruskal–Wallis H Test, a non-parametric alternative to ANOVA, was employed to compare the precision of diagnostic methods for non-ruptured cases and evaluate associations involving continuous variables. Approval from an ethics committee was waived due to the retrospective nature of the study.

## 3. Results

A total of 62 case reports and case series were identified, encompassing 156 patients with Type IIIb endoleak. The three most common endografts involved were AFX (Endologix, Inc., Irvine, CA, USA), Zenith (Cook Medical, Bloomington, IN, USA), and Talent (Medtronic, Minneapolis, MN, USA). The endoleaks were diagnosed at a median of 48 (0–216) months. In total, 35 cases (22.4%) presented with rupture, 8 cases (5.1%) had no presentation details mentioned, and 113 cases (72.4%) presented with intact aneurysms (Table 1). A total of 25 patients (16%) had no increase in aneurysm sac diameter, diameter increase was not mentioned in 46 cases (29.5%), and 85 cases (54.5%) presented with increased aneurysm sac diameter. Diagnoses were made by CT angiography in most cases (n = 69, 44.2%), by conventional angiography (n = 15, 9.6%), intraoperative diagnosis (n = 13, 8.3%), and Duplex ultrasound (n = 10, 6.4%). The rest of the cases were diagnosed intraoperatively after an unclear CT scan. There was a considerable number of cases where the method of diagnosis was unclear (n = 48, 30.8%).

The data show that CTA was the most precise and frequently used method for diagnosing both non-ruptured and ruptured aneurysms. For non-ruptured aneurysms, CTA diagnosed 55 cases, followed by unknown methods with 32 cases, angiography with 15 cases, DUS with 10 cases, and intraoperative methods with 9 cases. For ruptured aneurysms, unknown methods were used in 16 cases, CTA in 14 cases, and intraoperative methods in 4 cases. Notably, angiography and DUS were not used to diagnose any ruptured aneurysms.

Most fabric tears were located in the flow divider (n = 68, 43.6%) and the main body (n = 44, 28.2%). Ninety-four patients (60.3%) were treated with endovascular techniques, twenty patients (12.8%) underwent EVAS (EVAS, Endologix Inc. Nellix, Irvine, CA, USA), and thirty-eight patients (24.4%) were treated with open surgery. Four patients (2.6%) refused therapy or were physiologically not amenable to any type of repair, and two patients (1.3%) were treated by a combination of endovascular and open repair, with the true nature of the therapy not clearly described.

Our findings indicate statistically significant associations between increasing diameter and leaks in the main body (*p* = 0.01) and the flow divider (*p* = 0.005).

Nineteen patients had endoleak-related mortality (12.2%). In a total of 30 cases, Type IIIb-related mortality was not mentioned (unknown). Overall, 107 patients (68.6%) were successfully treated. Our findings indicate a statistically significant association between leaks in the main body and rupture (*p* = 0.0047), as well as leaks in the flow divider and rupture (*p* = 0.002). Our findings revealed statistically significant associations between Talent (Medtronic) stent grafts and leaks in the main body (*p* = 0.001), Vanguard (Boston Scientific, Marlborough, MA, USA) stent grafts and leaks in unknown locations (*p* = 0.001), Vanguard (Boston Scientific) stent grafts and leaks in the flow divider (*p* = 0.005), AFX (Endologix, Irvine, CA, USA) stent grafts and leaks in the flow divider (*p* = 0.01), and Incraft (Cordis, Vienna, Austria) stent grafts and leaks in the right limb (*p* = 0.04). No other significant associations were found.

One patient with a Stentor (MinTec, Buckinghamshire, UK) graft implanted in 1998 experienced a rupture after 9 months. Three patients with Vanguard (Boston Scientific) grafts implanted in 2001 experienced ruptures at 18, 24, and 28 months, respectively. Six patients with Zenith (Cook, Bloomington, IN, USA) grafts implanted in 2008, 2012, and 2014 had ruptures between 30 and 84 months post-implantation. Four patients with Excluder (W.L. Gore, Flagstaff, AZ, USA) grafts implanted in 2013, 2019, and 2022 had ruptures at 10, 60, and 72 months. Six patients with Talent (Medtronic) grafts implanted in 2014, 2018, and 2021 experienced ruptures between 60 and 132 months. Eight patients with AFX (Endologix) grafts implanted in 2016 and 2022 had ruptures ranging from 11 to 51 months. Seven patients with Endurant (Medtronic) grafts implanted in 2015, 2016, 2019, and 2021 experienced ruptures at 6, 14, 48, and 72 months. Additionally, three patients with unknown graft types experienced ruptures occurring up to 96 months post-implantation.

The predominant repair technique employed was endovascular relining, performed in 101 patients (65.7%). This was followed by relining with the Nellix (EVAS) prosthesis in 20 patients (12.8%). Open surgical repair was the treatment of choice for 29 patients (18.6%). Four patients (2.6%) opted for conservative therapy either due to personal preferences or because they were physiologically unfit for any surgical intervention. Since we could not find sufficient mortality data from open and endovascular repairs, a comparison between the two methods was not possible.

Only one publication mentioned a potential Type IIIb endoleak caused by fabric erosion due to infection [12].

## 4. Discussion

Transgraft endoleaks are considered high-flow, high-pressure endoleaks and should be managed similarly to Type I endoleaks, necessitating immediate intervention upon diagnosis. The primary issue with Type IIIb endoleaks is the scarcity of data available and the lack of a standardized reporting framework. Consequently, there is significant variability in reporting between case reports and case series. Detailed anatomical information about aneurysms is generally lacking. Gennai et al. [13]. and Hoshina et al. [14] reported anatomical information on the aortic neck. Only in two publications was adherence to the instructions for use (IFUs) of endoprostheses mentioned. Fujimura and colleagues reported seven cases of Type IIIb and three cases of Type V endoleaks, with IFU violations observed in 40% of all EVAR procedures conducted [15]. McWilliams and colleagues stated that the endoprosthesis was implanted according to the instructions for use [16].

We can subclassify Type IIIb endoleaks into two categories according to the definitions in the case reports and case series: immediate and late types. The immediate type can be caused by aggressive ballooning, implantation of an aortic cuff or a stainless steel Giant Palmaz Stent [17,18,19], and manufacturing processes. The occurrence of Type IIIb endoleak was attributed to the loading of the endoprosthesis into its sheath by two studies [17,20]. Lemmon et al. [21] documented a case of Type IIIb endoleak in a study where a Type Ia endoleak was treated using proximal aortic extensions (Vela, Endologix). Seike et al. [22] reported prior intervention for a Type Ia endoleak using a proximal aortic cuff before the manifestation of a Type IIIb endoleak. Reijnen et al. [23] presented a similar scenario, where a Type Ia endoleak was treated with a proximal cuff prior to the development of a Type IIIb endoleak. Kwon et al. [24] indicated that additional procedures included the repair of kinked limbs, Type Ia endoleak repair with Palmaz stent or proximal cuff deployment, Type Ib and Type IIIa endoleak repair, and additional balloon angioplasty of limbs before the onset of a Type IIIb endoleak. Dayama et al. [25] reported that a Type Ia endoleak was addressed through aortic angioplasty, followed by the placement of a Palmaz stent before the occurrence of a Type IIIb endoleak. Fujimura et al. [15] reported an equal incidence of Type Ia and Type III endoleaks, with seven cases of Type IIIb and one case of Type IIIa endoleak.

Late-occurring Type IIIb endoleaks can be attributed to fabric fatigue and, consequently, erosion and stent fractures [17,26,27]. The stress caused by each pulse may lead to microdamage to the endograft stents, which might eventually fracture.

Jacobs et al. [28] described mechanical failure modes after examining endografts under an electron microscope. Stent fractures, fabric fatigue, and suture breakage were identified as the main failure modes. Stent fractures due to the effects of corrosion were identified in Stentor grafts (MinTec, Bahamas); however, significant corrosion in other nitinol stents could not be confirmed with electron microscopic studies. This can be attributed to the improved manufacturing of modern stents with micropolishing and coating [28].

In the Stentor (MinTec, Bahamas) and Vanguard (Boston Scientific, Marlborough, Mass) devices, the stent is attached to the graft only at the proximal and distal ends. Furthermore, polypropylene sutures are used to assemble the devices, allowing for continuous motion between the stent and the graft. Micromotion of the individual nitinol stents causes friction and wear of the sutures, ultimately leading to suture fracture and stent row separation [28].

Several known issues related to graft design and fabric have already been published. The Type IIIB endoleak issue of the Endologix (Irvine, CA, USA) AFX device (2009–2012) with STRATA fabric (ePTFE) is well documented, as highlighted by Lemmon et al. [29], which led to the recall of the device by the FDA. In 2014, the AFX endograft was modified to include Duraply-fabric, and a reduction in Type IIIB endoleaks has been documented. We found that AFX (Endologix) stent grafts have a statistically significant higher incidence of Type IIIb endoleaks in the flow divider (*p* = 0.01). This suggests that specific stent graft types may be more prone to leaks in certain anatomical locations, underscoring the importance of careful selection and monitoring of stent grafts based on individual patient anatomy and the type of graft used. The anatomical design of these grafts leads to greater contact between the graft and the aortic surface. Our findings, which are consistent with other studies, indicate that the primary cause of Type IIIb issues with AFX endografts is related to the fabric rather than its anatomical fixation design [13].

Studies on the biodegradability and biostability of polyethylene terephthalate (e.g., Dacron; Invista, Kennesaw, Ga) have shown that the loss of burst strength first appears after 10 years, which exceeds the diagnosis of Type IIIb endoleaks after the implantation of the endograft. Another study showed that Polytetrafluoroethylene (e.g., Gore-Tex; W. L. Gore) is biostable and does not undergo biodegradation [30]. Since data show the occurrence of endoleaks at a median of 48 months, this indicates that the occurrence of fabric tears cannot solely be attributed to the fabric itself but rather to a multifactorial etiology.

Maleux et al. [11] demonstrated a reduced incidence of Type III endoleaks in third-generation endografts compared to first- and second-generation grafts, although this difference was not statistically significant (*p* = 0.38). The cause of Type III endoleaks in first- and second-generation endografts was attributed to fabric tears in 50% of cases, while the cause of recurrent Type III endoleaks was due to fabric tears in 80% of cases [11].

As can be seen, improvements in device manufacturing and the materials and fabrics of the grafts have impacted the occurrence of Type IIIb endoleaks but have not completely eliminated them. The difference in the time of diagnosis of Type IIIb endoleaks between first- and second-generation endografts and third-generation endografts is not significant (3.87 vs. 5.92 years, *p* = 0.148) [11].

**Figure 1:**
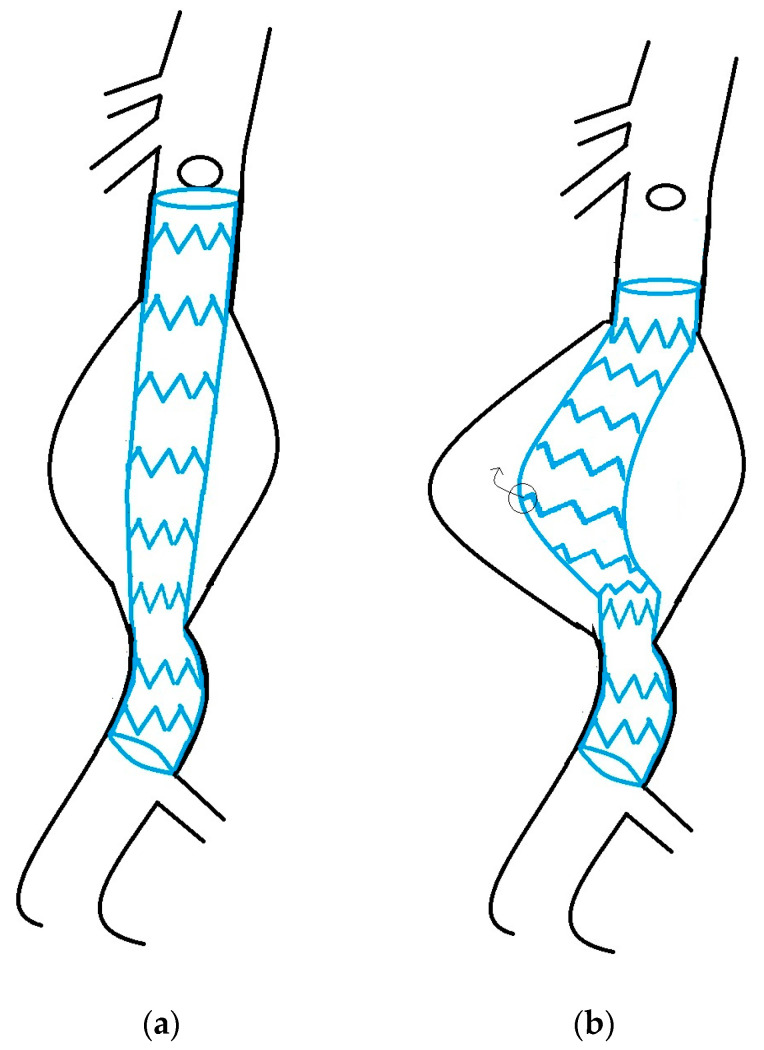
(**a**) before migration; (**b**) after Prosthesis migration from any cause (e.g., endoleak Type Ia or Ib) and aneurysm sac expansion can lead to redundancy of the endograft, resulting in unfavorable conformations that increase stress on stents and fabric. The arrow depicts the outward displacement force of the Z-stent against the fabric, creating a force favorable for developing a fabric tear).

Endografts are designed and improved to withstand the physical forces acting on them. However, aortic disease is an active process, and with the progression of aneurysmal disease, conformational changes in the stentgraft might occur. This may lead to exposure to much higher physical stress than initially intended by their design. A Type IA or IB endoleak, which leads to an increase in aneurysm size and endograft migration that causes a downward and anterior displacement of the graft, can result in kinking. This kinking might lead to increased shear stress and/or increased outward pull on the metal stent, acting on the fabric and potentially leading to the formation of fabric defects. Excessive kinking of the limbs, distal migration of the main body, forward bending, and the occurrence of kinks were suggested as reasons for Type IIIb endoleak in five studies [14,17,18,31,32] (Figure 1).

Our independent assessment of published angiograms in the case reports and series revealed a non-negligible amount of significant angulations and kinks of either the prosthesis limbs or the main body. Unfortunately, these were not mentioned in the reports themselves and cannot be quantified.

Additionally, even in the absence of migration, a large aneurysm sac provides space for the stentgraft to displace, especially in the presence of Type II endoleaks. Lemmon et al. [29] found no relationship with device implantation, surgeon, completion balloon angioplasty, or aneurysm morphology except for aneurysms greater than 65 mm in size between AFX and non-AFX grafts. A case series by Paraskevas reported a median aortic diameter of 94.5 mm (range: 55–120 mm) at the diagnosis of Type IIIb endoleak [33]. Hoshina [14] described anterior migration of the prosthesis associated with a Type Ia endoleak, which subsequently led to a Type IIIa endoleak.

Cross-limb conformation might also cause increased shear stress. The conformation of the limbs leading to Type IIIb endoleak was not mentioned in any studies. However, a careful assessment of published angiography images indicated that the crossed limb configuration, also known as the “ballerina configuration”, was not uncommon. A Type IIIb endoleak due to this configuration was confirmed in an explanted endograft [34]. 

Furthermore, increasing the stiffness of a certain part of the endograft by relining the limb or with a cuff might create a pivot-like mechanism, resulting in stiffer and less stiff parts of the endograft. This can lead to recurrent fabric defects by creating more mobile and less mobile parts of the endograft. Another important contributor can be the calcium content and hostile features of the neck, as described in one study [14], against which the endograft is continuously in contact and rubbed.

**Figure 2:**
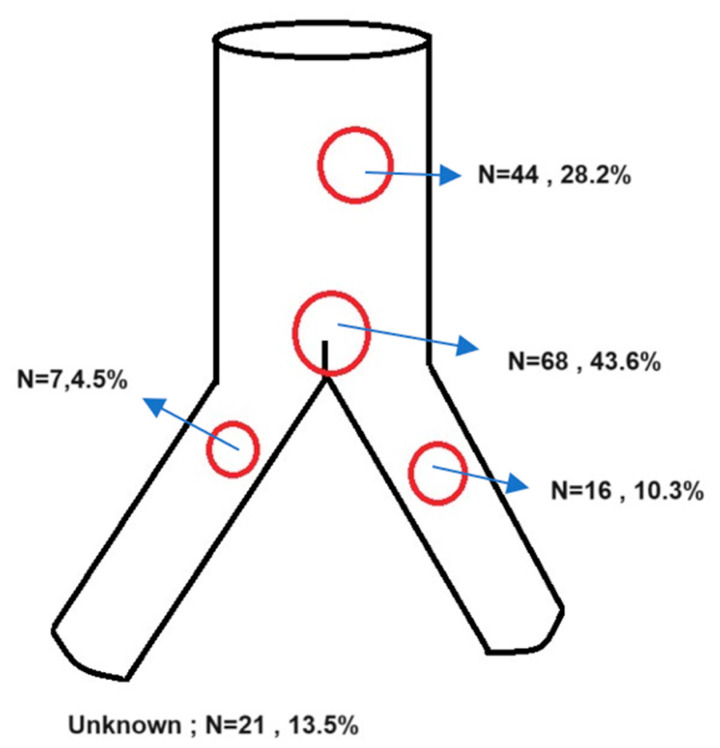
Location of the fabric defect/Type IIIb endoleaks. This figure illustrates the distribution of Type IIIb endoleaks in the cohort. Of the total endoleaks, 44 (28.2%) were located in the main body of the endograft, while 68 (43.6%) were found precisely at the flow divider. Additionally, 7 endoleaks (4.5%) occurred in the right limb, and 16 endoleaks (10.3%) were identified in the left limb.

The possible association of the physical forces acting on the stentgrafts can be simulated using computational fluid dynamics (CFD) models. Liu et al. [35] investigated the crossed limb conformation in endografts and hypothesized that if helical flow can be created with crossed limb conformation, it could reduce graft limb occlusions. They found that the crossed limb strategy can be beneficial due to creating helical flow patterns, which might reduce limb occlusions, but unfavorable hemodynamics were observed with TAWSS, OSI, and RRT measurements. To define briefly, TAWSS (Time-Averaged Wall Shear Stress) is a measure of the shear stress exerted by the blood flow on the vessel walls, averaged over a cardiac cycle. OSI (Oscillatory Shear Index) quantifies the directional changes in wall shear stress over a cardiac cycle. High OSI values are often found in regions where the flow is disturbed or oscillates significantly. RRT (Relative Residence Time) assesses the time that fluid particles spend in a given region of the flow domain. Higher RRT values indicate longer residence times. Liu concluded that the risk of migration was increased, and ribbon areas were created in the limbs of the graft, which might increase thrombus formation and lead to occlusion in crossed limb configuration [35]. We see an interesting coincidence in the locations of the fabric tears (Figure 2), matching the areas with unfavorable measurements of TAWSS, OSI, and RRT. Another study by Lu et al. [36] also found a high degree of correlation between wall stress and endoleak formation. Although the type of endoleak was not mentioned, the location of the fabric tears matched exactly to high-stress areas on the 3D plots as well. Another study indicated that the higher helicity and improved wall shear stress (WSS) distribution observed in the cross-limb configuration suggest better short-term resistance to flow-related thrombosis. However, this configuration may also lead to long-term fatigue issues for stent grafts compared to the direct configuration [37]. In the literature, we could not identify any computational fluid dynamics model directly addressing Type IIIb endoleaks.

As we can see, the locations of fabric tears are in the locations with high TAWSS, OSI, and RRT measurements, especially with the crossed limb configuration [35]. We believe that the redundant conformation of the endograft might also increase shear stress and can lead to fabric defects due to exposure to excessive shear stress.

Another important theme is the diagnosis of Type IIIb endoleaks. The results showed that the majority were diagnosed using CT angiography, but there was a significant number of cases where the method of diagnosis was unclear or unknown. The true incidence of Type IIIb endoleaks might, therefore, be much higher than expected, as only 20% of Type IIIb endoleaks were detected with CT angiography, according to one study [8]. Nishibe et al. [38] suspected that a certain number of Type V endoleaks might actually be Type IIIb endoleaks.

A limitation of this study is the limited number of publications, discrepancies in reporting of Type IIIb endoleaks, a lack of reports from newer-generation endografts, and publication bias.

## 5. Conclusions

Type IIIb endoleaks are high-pressure endoleaks which can lead to aneurysm sac expansion and rupture; therefore, they must be treated if the diagnosis is certain. Early Type IIIb endoleaks can be attributable to excessive ballooning, placement of cuffs, or Giant Palmaz stents, while late Type IIIb endoleaks are a result of material fatigue. However, material fatigue can be caused by manufacturing issues, material/stent design, or physical forces acting on the endograft. Conformational changes in the endograft can lead to redundant configurations, which might cause increased stress acting on the endograft far beyond its design limits.

In conclusion, our findings underscore the critical importance of strictly adhering to the instructions for use (IFUs) provided by the manufacturers. We recommend implementing a closer follow-up protocol and additional diagnostics for patients exhibiting conformational changes or redundancy of the endografts, especially if the aneurysm is re-expanding or not shrinking after repair. Additionally, we suggest more vigilant monitoring for patients who have undergone EVAR for large aneurysms. These measures are essential to ensure the long-term success and safety of endovascular aneurysm repair.

## Figures and Tables

**Table 1:** Results.

	N (%)
Type of Endoprothesis	
AFX-Strata (Endologix), AFX-Duraply (Endologix, Inc., Irvine, CA, USA)	54 (34.6%)
Zenith Flex (Cook Medical, Bloomington, IN, USA)	31 (19.9%)
Talent (Medtronic, Minneapolis, MN, USA)	20 (12.8%)
Endurant (Medtronic, Minneapolis, MN, USA)	15 (9.6%)
Vanguard (Boston Scientific)	10 (6.4%)
Unknown	8 (5.1%)
Excluder (W.L. Gore, Flagstaff, AZ, USA)	5 (3.2%)
Nellix (Endologix Inc. Nellix, Irvine, CA, USA)	3 (1.9%)
Ancure (Guidant Cardiac and Vascular Division, Menlo Park, CA, USA)	3 (1.9%)
Anaconda (Sulzer Vascutek, Austin, TX, USA)	3 (1.9%)
Stentor (Boston Scientific, Natick, MA, USA)	1 (0.6%)
Incraft (Cordis)	1 (0.6%)
Aneuryx (Medtronic, Minneapolis, MN, USA)	1 (0.6%)
AegisB (Endovastec, Shanghai, China)	1 (0.6%)
Diagnosis	
CT-Angiography	69 (44.2%)
Unknown	48 (30.8%)
Angiography	15 (9.6%)
Intraoperative	13 (8.3%)
Duplex ultrasound	10 (6.4%)
Diagnosis after Implantation, median months (max–min)	48 (0–216)
Rupture	
Yes	35 (22.4%)
No	113 (72.4%)
Unknown	8 (5.1%)
Diameter Increase at the time of Diagnosis	
Yes	85 (54.5%)
No	25 (16%)
Unknown	46 (29.5%)
Location of the Fabric tear	
Flow Divider	68 (43.6%)
Main body	44 (28.2%)
Left Limb	16 (10.3%)
Right Limb	7 (4.5%)
Unknown	21 (13.5%)
Type of Repair	
Open	38 (24.4%)
Endovascular (Reline, Cuff)	94 (60.3%)
Endovascular (EVAS, relining with Nellix system, Nellix Reline, Palo Alto, CA, USA)	20 (12.8%)
Conservative (no consent or not fit)	4 (2.6%)
Mortality	
Yes	19 (12.2)
No	107 (68.6)
Unknown	30 (19.2)

## Data Availability

The original contributions presented in the study are included in the article; further inquiries can be directed to the corresponding author.
